# Dose–effects in behavioural responses of moths to light in a controlled lab experiment

**DOI:** 10.1038/s41598-023-37256-0

**Published:** 2023-06-26

**Authors:** Annika Jägerbrand, Petter Andersson, Maria Nilsson Tengelin

**Affiliations:** 1 Department of Electrical Engineering, Mathematics and Science, Faculty of Engineering and Sustainable Development, University of Gävle, Gävle, Sweden; 2 Calluna AB, Hästholmsvägen 28, 131 30 Nacka, Sweden; 3 Department of Measurement Science and Technology, RISE Research Institutes of Sweden, Borås, Sweden

**Keywords:** Ecology, Animal behaviour, Entomology

## Abstract

Insects play a critical role in providing numerous ecosystem services. However, insect diversity and biomass have been declining dramatically, with artificial light being suggested as a contributing factor. Despite the importance of understanding the dose–effect responses of insects to light emissions, these responses have been rarely studied. We examined the dose–effect responses of the greater wax moth (*Galleria mellonella* L.) to different light intensities (14 treatments and a dark control) by observing their behavioural responses in a light-tight box equipped with a LED light source (4070 K) and infrared cameras. Our findings reveal dose–effect responses to light, as the frequency of walking on the light source increased with higher light intensity. Additionally, moths exhibited jumps in front of the light source and jump frequency increased with light intensity. No direct flight-to-light behaviour or activity suppression in response to light was observed. Based on our analysis of the dose–effect responses, we identified a threshold value of 60 cd/m^2^ for attraction (walking on the light source) and the frequency of jumps. The experimental design in this study offers a valuable tool for investigating dose–effect relationships and behavioural responses of various species to different light levels or specific light sources.

## Introduction

Insects represent the most species-rich animal group on the planet and provide crucial ecosystem services, including pollination, decomposition, soil formation and biological control of pest species^1^. Numerous studies have reported significant declines in insect diversity and biomass^2–6^. These declines have been attributed to a variety of factors, including habitat destruction and degradation, climate change, changes in land use and habitat fragmentation^3,7^, and the potential effects of artificial light at night^8,9^.

Many organisms, including animals and microorganisms exhibit phototaxis, a behaviour characterized by movement towards or from light (e.g.,^10,11^). Nocturnal insects, in particular, are commonly attracted to light and can be found circulating streetlights or resting below light sources on the ground^12,13^ or building facades. Therefore, many studies have taken advantage of this positive phototaxis by using light traps in studies of nocturnal lepidopterans (e.g.,^14–16^). However, concerns have been raised about the potential impact of increased light pollution on nocturnal animals, including disruptions to their circadian rhythms and populations^8,17^.

While many studies have examined the effects of artificial light at night on insects under field conditions by comparing light sources or light intensities (e.g.,^18–25^), controlled experimental investigations of dose–effect responses to light intensity have been rare^26^. However, understanding such dose–effect responses is crucial for predicting the outcomes of ecological perturbations. For example, dose–effect responses are commonly observed in studies of insect behaviour as a function of the release rates of semiochemicals, such as pheromones and kairomones^27–29^.

To our knowledge, no previous study has comprehensively examined the dose–effect responses to light of nocturnal insects under controlled conditions. In this study, we present the dose–effect responses in activity (attraction and behavioural) of a nocturnal moth in a controlled lab experiment. Our study aimed to investigate whether dose–effect responses to light exist in these insects. We therefore developed a novel light-tight box in which it was possible to precisely control light conditions and monitor insect behaviour. The main idea behind this design was to expose the insects to controlled levels of light and darkness, eliminating any potential confounding factors, and observe their responses.

## Methods

### Study organism

The greater wax moth (*Galleria mellonella* L.) was selected as the study organism. This species is a worldwide distributed nest parasite of the honeybee (*Apis mellifera* L.). It is recognized as an economically important pest species due to its nesting behaviour in beehives and the larvae’s consumption of honeycombs. In southern parts of Sweden, the greater wax moth is found in low abundance. It is known to be attracted to light in the adult stage^30^ and is regularly collected in light traps by Swedish field lepidopterists. The greater wax moth is easily reared under lab conditions, possesses a short generation time, and has been extensively used as a model organism in immunological and biomedical research^31^.

The species is commercially available in Sweden, where the larvae are sold as food for herptiles. For our research, we obtained moth individuals in the larval stage from two pet stores. Since we worked with commercially available moths, we lack detailed information about the breeding history or previous rearing conditions. Therefore, we cannot be certain that the moths in our study exhibit the exact same behaviors as their wild counterparts. However, by purchasing moth individuals from two different breeders, we aimed to minimize the influence of potential confounding factors. It is unlikely that moths from different breeders were reared under identical conditions.

The larvae were housed in plastic containers placed inside a lab box. To minimize exposure to ambient light conditions, the box lid was kept closed for most of the time. Since wild wax moth individuals spend their entire larval and pupal stage in beehives, we considered this dark environment to be representative of their typical conditions. To maintain a stable temperature for larvae development, a heating pad was placed inside the wooden box, maintaining a stable temperature of 29–30 °C, which is optimal for larval development^31^.

The larvae were provided with a mixture of honey and oats as their food source in the plastic containers. After pupation and emergence, newly emerged adult moths (imagos) were used for the experiment. Moths continuously emerged from pupae throughout the duration of the experiment and were generally no more than 24 h old when utilized.

### Experimental setup

**Figure 1:**
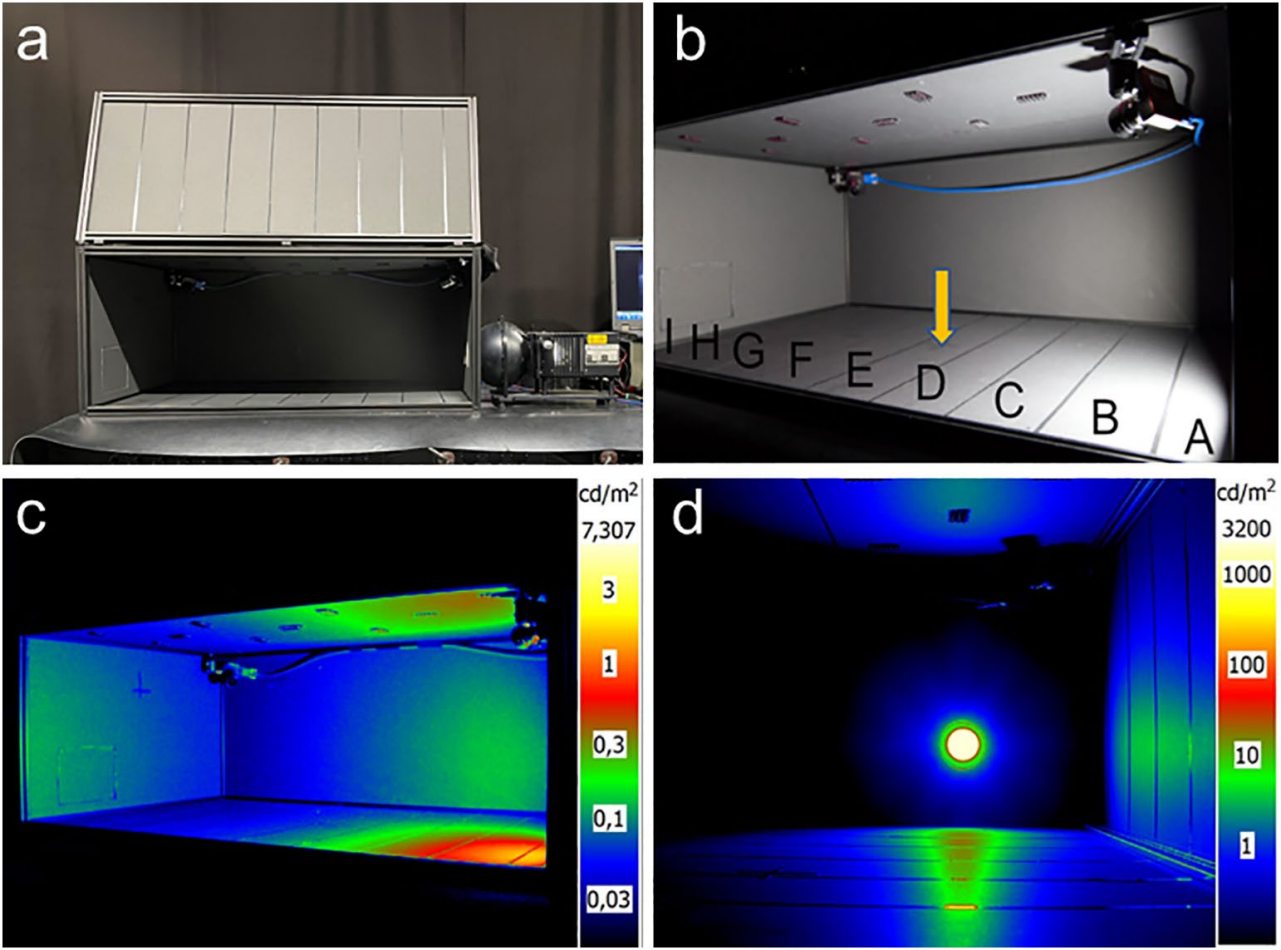
(**a**) Photograph of the box and the luminance source set-up; (**b**) Photograph of the interior of the box showing the position of infrared LEDs in the ceiling, cameras in opposing top corners, the luminance source on the right side, designated zones A–I used for determining the spatial position of the insects within the box, the yellow arrow depicting the illuminance measurements taken at the center of the box; (**c,d**) Luminance distribution within the box.

For the experiments, we constructed a rectangular light-tight box (Fig. 1) with a light source positioned on one of the short sides. The dimensions of the box were 60 × 45 × 90 cm (width × height × length). To monitor the movement of insects inside the box, we utilized two monochrome cameras with wide-angle objectives (Thorlabs model CS165MU/M Zelux® 1.6 MP Monochrome CMOS Camera, 1/2.9" sensor size equipped with Thorlabs model MVL5WA Navitar lens, 4.5 mm fixed focal length, f/1.4). These cameras were placed in opposite corners of the box to maximize coverage (Fig. 1b). To provide visibility, infrared LEDs (940 nm) were installed in the ceiling of the box. Since the radiation emitted by these infrared LEDs falls outside of the visible range of most insects^32,33^, it was presumed to have no disruptive effect on the insects in this experiment.

To ensure minimal reflections and optimal recording conditions, the walls of the box were constructed using a material that had diffuse and low reflectance (5%) within the visible range while offering high reflectance (> 60%) at 940 nm. Consequently, no noticeable reflections from the light source were observed on the walls. Simultaneously, the radiation emitted by the infrared LEDs in the ceiling was evenly distributed, resulting in a well-illuminated interior during the recordings. This allowed us to obtain high-quality film sequences capturing the movements of the moths. To determine the moths’ position accurately, a grid was drawn on the floor and on one of the side walls of the box (Fig. 1b).

**Figure 2:**
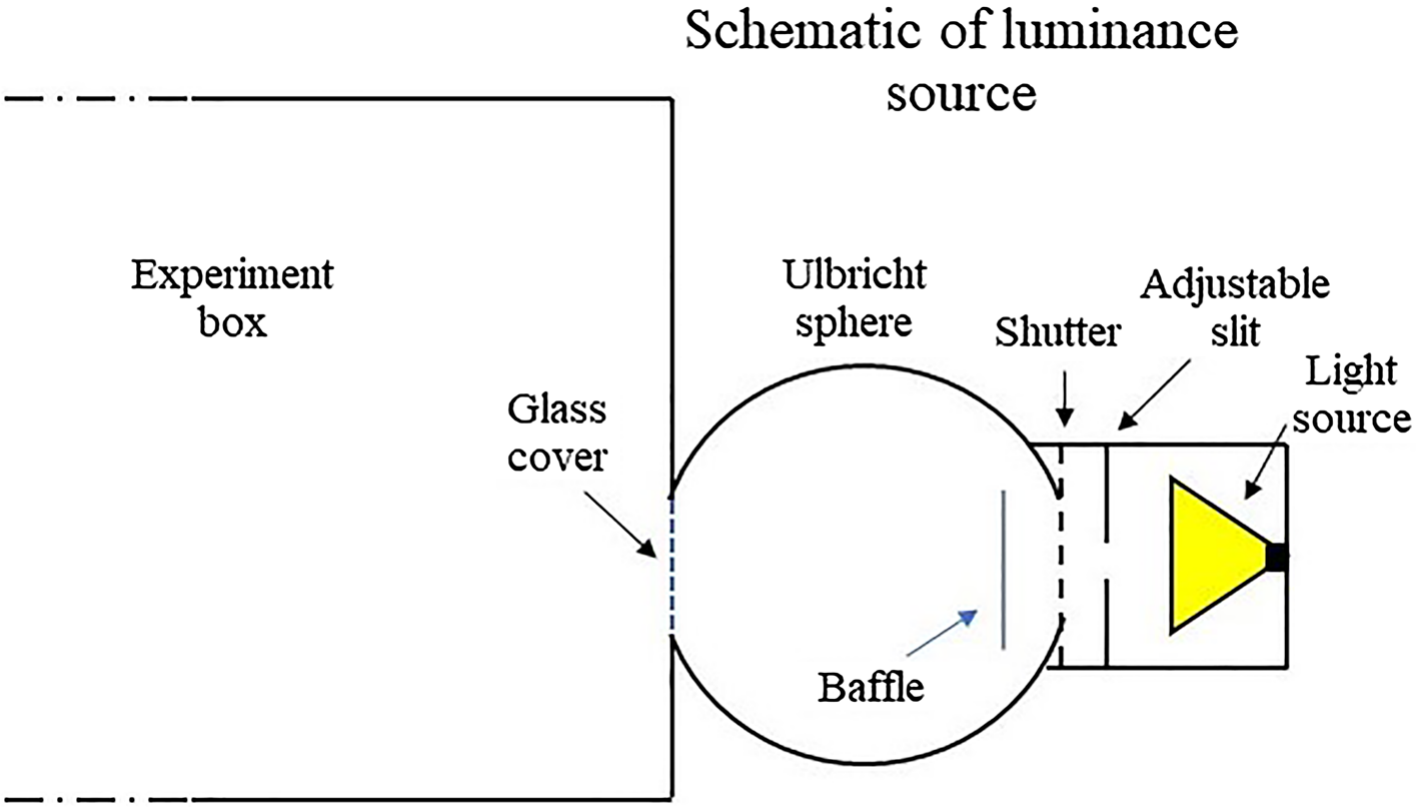
Schematic picture of the luminance source.

For the light source, we utilized a standard 7 W white-light LED with a correlated colour temperature of 4070 K. It was installed in a conventional sphere-based luminance source (Figs. 1, 2). To prevent any insects from entering the sphere, we placed a glass cover over its opening facing the box. A mechanical shutter was employed to control the on/off state of the light input to the sphere. This enabled us to achieve stable light levels without requiring any stabilization time. In addition, the luminance source featured an adjustable slit positioned between the light source and the sphere. This allowed for continuous adjustment of the light intensity, ranging from maximum (with the slit fully open) to complete darkness (closed slit). As the adjustment was performed without dimming the LED, the experiment maintained the exact spectral power distribution of the light source consistently (see Supplementary Fig. S1 for the spectral power distribution of the light source).

### Experimental procedures

Prior to experimental trials, the insect containers were kept dark for at least 24 h. The experiments were conducted at room temperature, approximately 23 °C. Throughout the experiments, the room was maintained in darkness, except for a small red light used to guide the transfer of insects from the container to the test box. One moth at a time was introduced into the test box, ensuring that the shutter to the light source remained closed, keeping the insect in darkness. After a duration of 60 s, the shutter to the luminance source was opened, exposing the moth to a predetermined light level. The light levels were randomly varied across trials. Subsequently, the movement and behaviour of the moth were recorded for a period of 10 min following the opening of the shutter. For the dark control trials, the shutter remained closed throughout the entire 11-min test to maintain a consistent time period in the light treatment trials. To minimize odor contamination inside the box, all surfaces were cleaned with ethanol (70%) between trials. The maximum luminance level tested was 3,200 cd/m^2^ with a range down to 1.6 cd/m^2^ (Table 1). Illuminance measurements were taken vertically and horizontally at the centre of the box (Table 1) to assess the light exposure experienced by the released moths. Illuminance and luminance were measured using a calibrated photometer (Hagner Universal photometer S4).

**Table 1:** Source luminance and illuminance levels at the vertical, horizontal, and back wall in the light-tight box for the 14 different light treatments included in the test.

Source luminance (cd/m^2^)	Illuminance (vertical in the centre of the box, height 2.8 cm), lx	Illuminance (horizontal 2 cm above the floor in the centre of the box), lx	Illuminance (vertical, back wall centre, height 2.8 cm), lx
3,200	17.7	2.92	6.30
2,240	12.4	2.04	4.41
1,280	7.08	1.17	2.52
640	3.54	0.584	1.26
320	1.77	0.292	0.630
160	0.885	0.146	0.315
80.0	0.443	0.0730	0.158
60.8	0.336	0.0555	0.120
40.0	0.221	0.0365	0.0788
28.8	0.159	0.0263	0.0567
19.5	0.108	0.0178	0.0384
16.0	0.0885	0.0146	0.0315
3.20	0.0177	0.00292	0.00630
1.60	0.00885	0.00146	0.00315

### Data and statistical analysis

Moth behaviour was analysed using two video recordings taken simultaneously from different angles during each experimental trial. Based on the moth behaviours observed in two pilot studies^34,35^ conducted prior to our experiment, we identified four specific behavioural responses for further analysis. First, we recorded the total duration in minutes that a moth was active, i.e., engaged in movement inside the box, during the trial. The purpose of this measurement was to investigate potential patterns of activity suppression in response to different light-level treatments. Second, we examined whether the light level treatment affected the moths' inclination to fly inside the box. This variable was categorized as a binomial response (yes/no). Third, we documented whether the moths displayed an attraction to the light source as a response to light treatment. We assessed this behaviour using two methods: (i) scoring the frequency of a moth walking on the light source during a trial, and (ii) recording the probability of walking on the light source as a binomial variable (yes/no). Finally, we examined the frequency of small jumps performed by the moths during the experimental trial. This jumping behaviour was frequently observed throughout the experiment and resembled small flips in various directions. We recorded the frequency of jumps executed during each trial and also noted the corresponding zone within the box (as depicted in Fig. 1b) to provide a rough measure of the spatial distribution of the jumps. All data is available in supplementary Datafile S1.

For all analyses, we examined the impact of the light-level treatment (source luminance measured in cd/m^2^ and log-transformed) on the various behavioural response variables using generalized linear models (GLMs). To analyse the active time (i.e., the percentage of total experimental time that the moth was in motion) we employed a GLM with a binomial error distribution. We used a quasibinomial error distribution since initial analyses indicated overdispersion in the data. Additionally, we analysed the probability of moths flying during the experimental trials as a binary variable (0/1). This analysis was conducted using a GLM with a binomial error distribution.

The frequency that the moths walked on the light source was examined using a GLM with a negative binomial error distribution, as preliminary analyses indicated overdispersion in the response variable. Additionally, we analysed the probability of moths walking on the light source as a binary variable (0/1) using a GLM with a binomial error distribution. Finally, the frequency of jumps performed by the moths during the experimental trials was analysed using a GLM. Initial analysis revealed overdispersion in this response variable, thus a negative binomial error distribution was used for this analysis as well.

Initially, we incorporated sex as an explanatory variable in all models and conducted an analysis of variance (ANOVA) to compare models with or without sex. However, the results consistently showed that sex did not have a significant impact on moth behaviour (*p* ≥ 0.12) and, therefore, it was excluded from further analysis (results not presented). All statistical analyses were conducted using R statistical Software 4.1.0^36^ with the add-on library MASS for the GLMs with a negative binomial error distribution.

## Results

The study examined the effects of 14 different light levels and a control condition in complete darkness on behaviour in a total of 96 trials. There were 17 replicates in the dark control group and 4–10 replicates for each light treatment (for more details, see supplementary Datafile S2).

**Figure 3:**
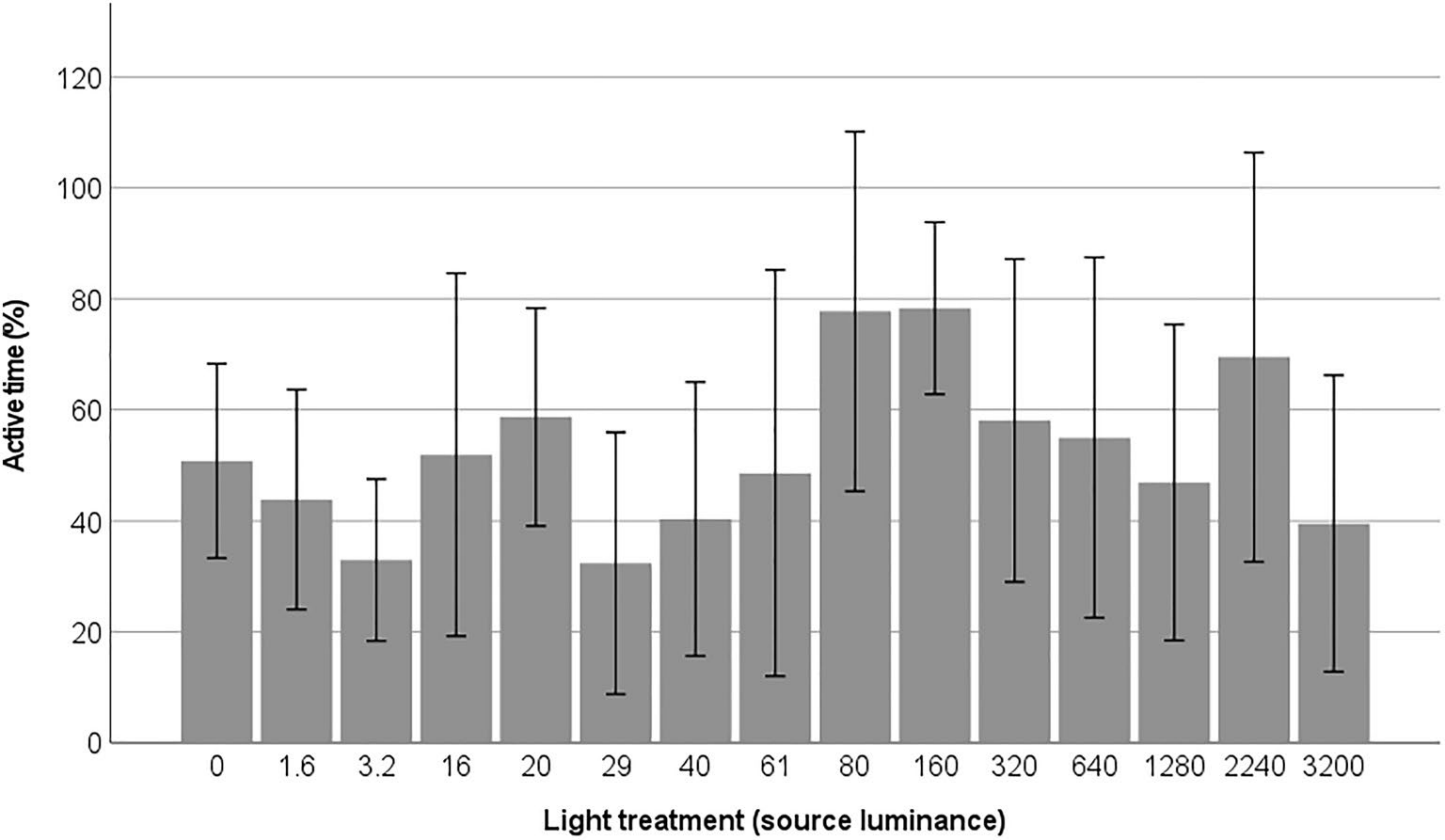
Percentage of active time in the light treatments (light source luminance in cd/m^2^). Mean values are shown with ± 2 standard errors.

**Figure 4:**
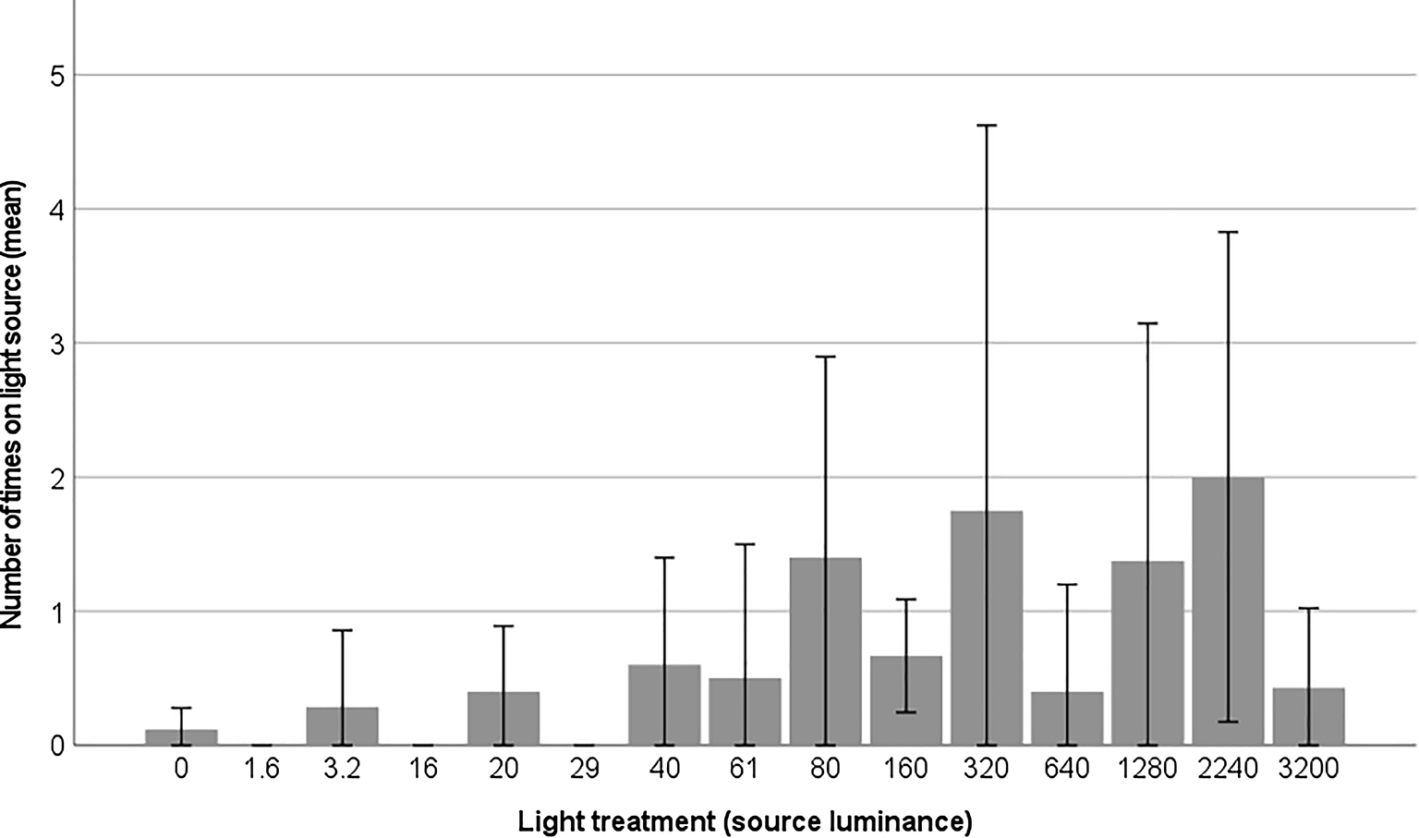
The frequency that the moths walked on the light source in the light treatments (light source luminance in cd/m^2^). Mean values are shown with ± 2 standard errors.

**Figure 5:**
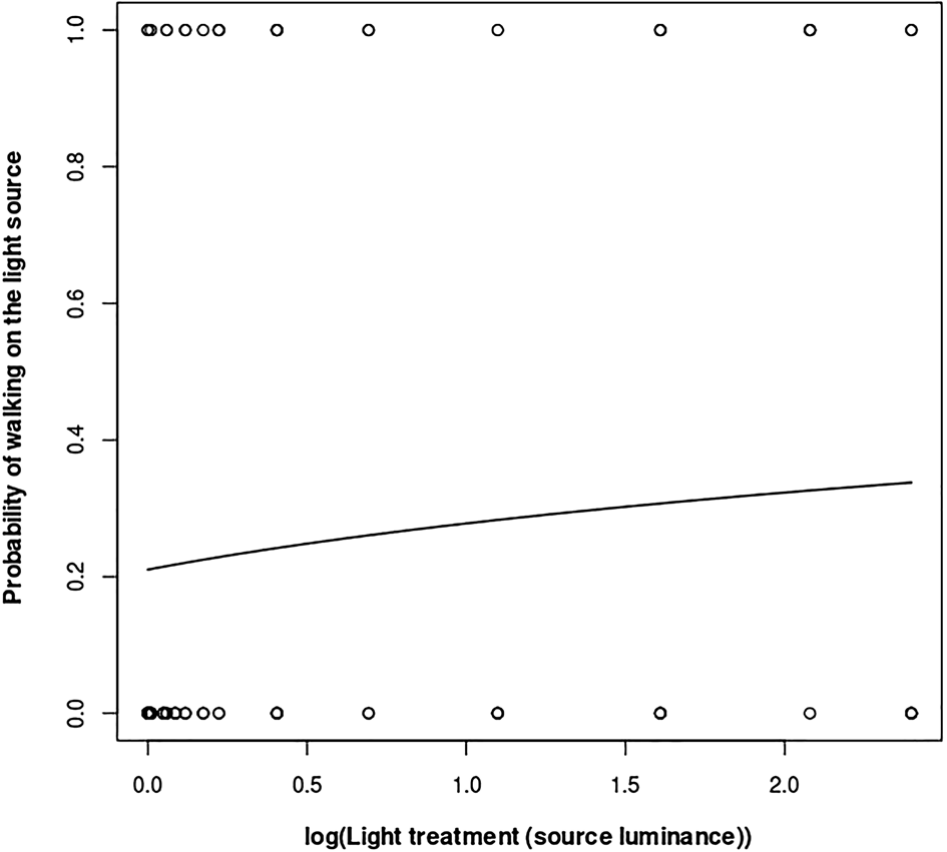
Probability of moths walking on the light source in the light treatments (light source luminance in cd/m^2^). The x-axis is displayed on a logarithmic scale.

The effects of the light-level treatment varied across different behavioural responses. Firstly, there was no evidence of activity suppression in the moths, as their activity levels were comparable in both the dark control trials and the light treatments (z = 0.17, n = 96, *p* = 0.87; Fig. 3). Flight activity was generally low, with only 26% of moth individuals flying during the trials. The probability of moths flying was not influenced by the light treatment (t = 1.36, n = 96, *p* = 0.17), indicating that light levels did not affect their flight activity. Additionally, we did not observe any direct flight-to-light behaviour in the moths. However, a significant dose–effect response was found between the light treatment and the frequency that the moths walked on the light source (z = 2.66, n = 96, *p* = 0.008; Fig. 4). The probability of walking on the light source during the experimental trials showed a nearly significant tendency to increase with light treatment (z = 1.91, n = 96, *p* = 0.057; Fig. 5).

**Figure 6:**
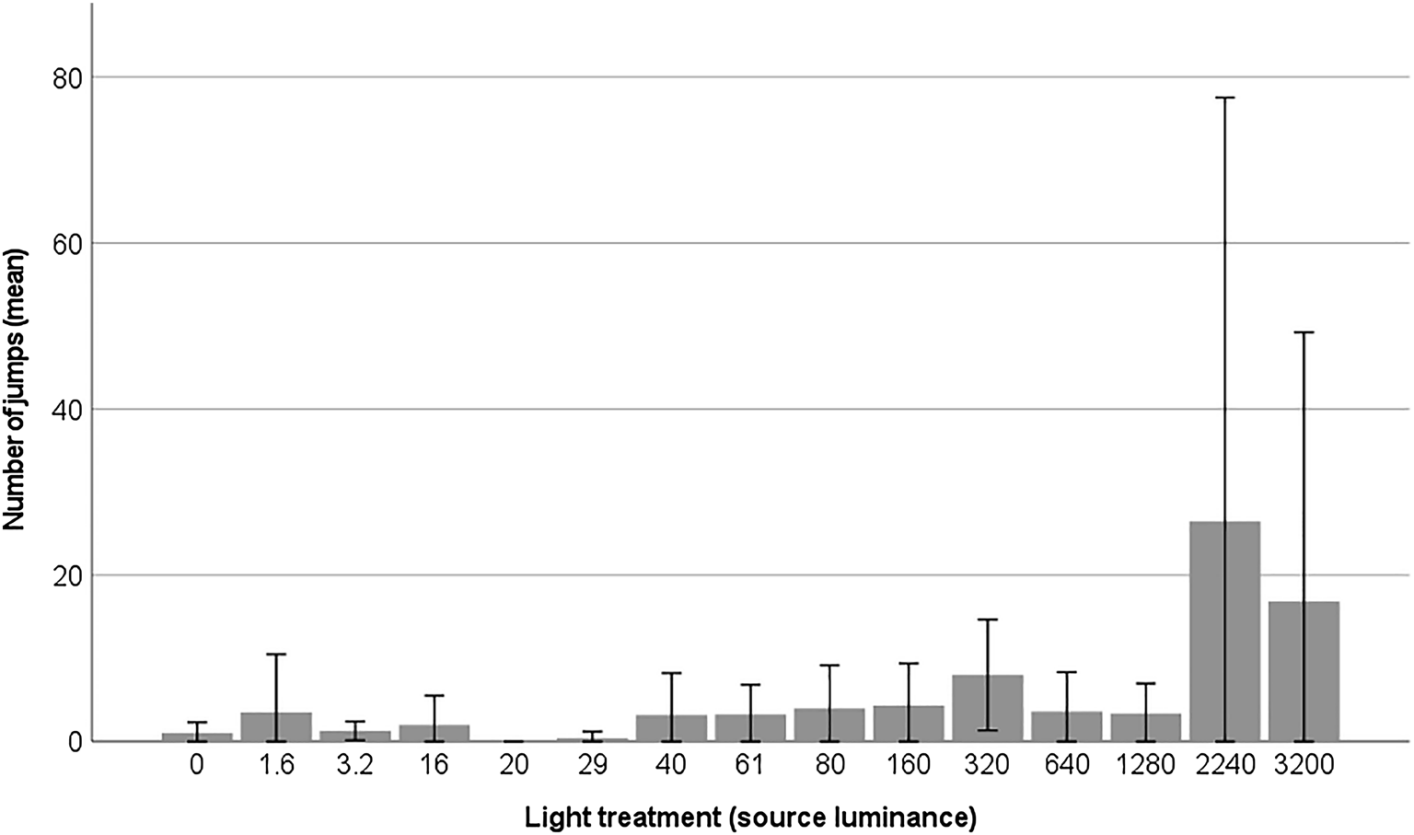
The frequency of jumps performed by moths in the light treatments (light source luminance in cd/m^2^). Mean values are shown with ± 2 standard errors.

**Figure 7:**
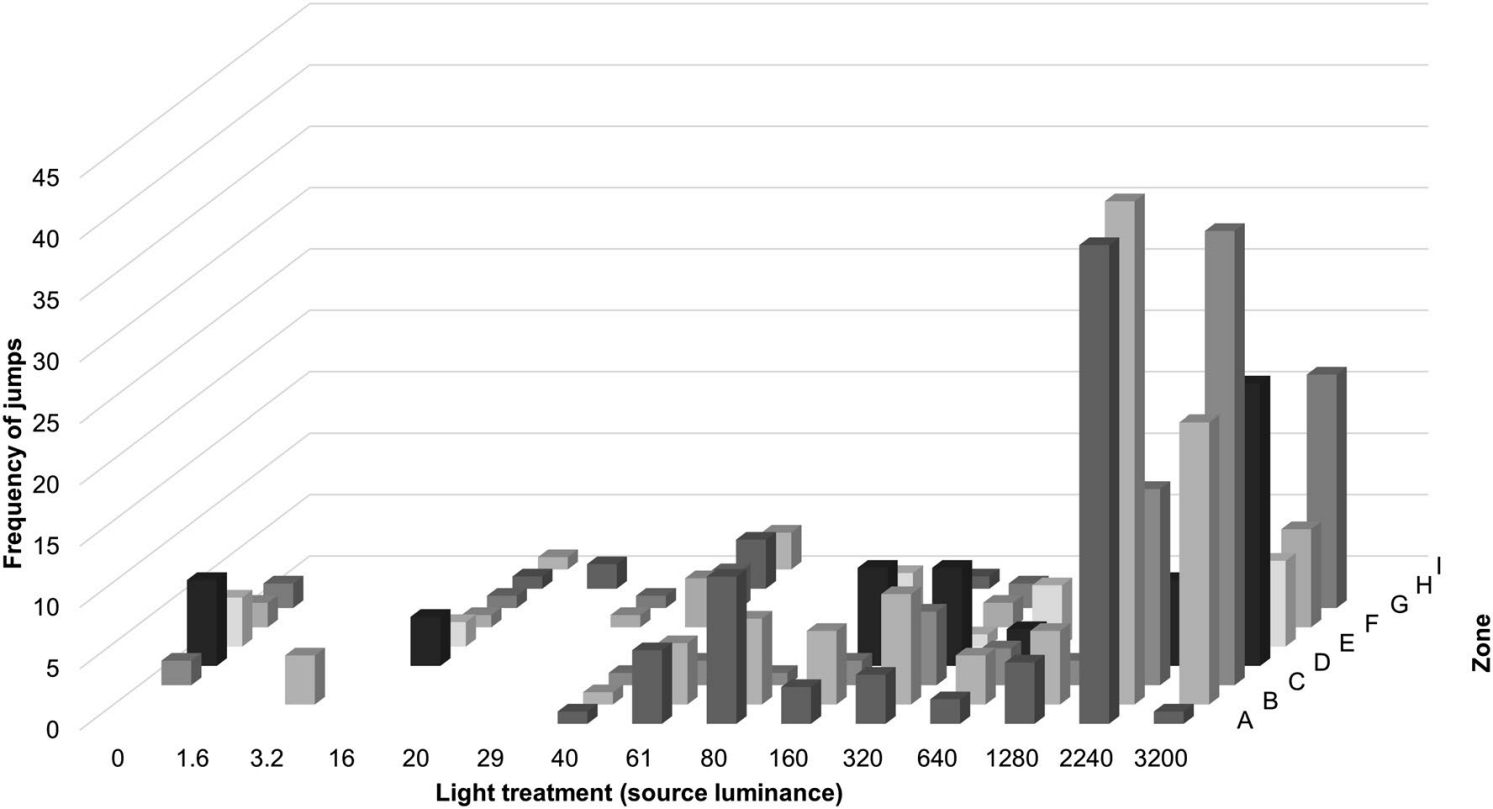
The frequency of jumps by moths in different zones within the light-tight box and under different light treatments (source luminance cd/m^2^). The jumps are represented for zones A–I as shown in Fig. 1 in the Materials and Methods section.

The frequency of jumps performed by the moths increased significantly with higher light levels, demonstrating a dose–effect response (z = 3.66, n = 96, *p* < 0.001; Fig. 6). Furthermore, the spatial distribution of these jumps within the experimental box revealed an interesting pattern, with moths exhibiting more jumps in the proximity of the light source, as depicted by the distribution of jumps among the zones within the box (Fig. 7).

A supplementary video file (S1) showcases the typical behavior of moths in the dark control condition, and walking on the light source and jumping in the light treatment 3200 cd/m^2^.

## Discussion

In this study, we investigated the dose–effect responses of moths to light treatments in a controlled lab experiment. Our findings revealed a significant dose–effect response in the frequency that the moths walked on the light source and the frequency of jumps observed during the experimental trials. Additionally, we observed a concentration of jumps in the area closest to the light source within the box. Interestingly, we did not observe significant effects of light treatment on any other response variables. This suggests that the mechanisms underlying the behavioural responses of nocturnal moths to artificial light may be much more complex than previously assumed. It is noteworthy that despite claims of suppressed activity in response to light being one of the most established responses of moths to artificial light^8^, we did not find any dose–effect responses in the overall activity of the moths.

One interesting pattern observed in the experiment was the slight increase and redistribution of jumps at approximately 60 cd/m^2^ with a more substantial increase between 1280 cd/m^2^ and 2240 cd/m^2^ (Fig. 7), indicating two threshold values for this behavioural response. These luminance values corresponded to 0.34 lx and 12.4 lx, respectively (measured vertically in the middle of the box). It was evident that the moths performed more jumps in the area with high luminance (Fig. 1c–d). In our experiment, the reflectance in the visible wavelength range of the walls of the box was very low (< 5 %). However, it cannot be ruled out that there is an impact of reflected light from walls, ceiling and floor as well as direct light from the light source. While it is generally assumed that direct light attracts insects, it is also known that reflected and scattered light in the sky, such as from sports arenas, can draw moths towards the light. For future studies, it would be intriguing to disentangle the effects of different levels of direct light versus reflected light from the perspective of moths. Obtaining such knowledge would enhance our understanding of how moths respond to specific light levels and the effects of reflected spill light in their surrounding environment. This knowledge is essential for developing effective mitigation measures and establishing threshold values. Unfortunately, guidelines for outdoor lighting and light pollution often overlook restrictions on reflected light^17^.

Our results further indicate a dose–effect response in the frequency that the moths walked on the light source (Fig. 3). In dark conditions and in a few light treatments (16 and 29 cd/m^2^), moths exhibited minimal activity on the light source. However, between 61–80 cd/m^2^, there was a noticeable increase in the frequency of moths walking on the light source. These luminance values correspond to 0.34 lx and 0.44 lx when measured vertically in the middle of the box. As a point of reference, illuminance during a full moon phase has previously been measured horizontally at 2 m to 0.2 lx at mid-latitudes^37^. It is likely that threshold values for light impacts or attraction of moths will be around or above natural moonlight levels. Although there are natural light sources with higher illuminance or luminance than moonlight at night (e.g., lightning strikes, forest fires), moths have limited exposure to them.

In our experiment, the frequency that the moths walked on the light source likely represented a behavioural expression of positive phototaxis or attraction to light. We observed a slight shift in behaviour at a luminance level of 61 cd/m^2^ in an increased frequency of jumps performed by the moths. While we defined activity as any form of movement rather than remaining stationary, it is important to note that activities can encompass various behaviours such as flying, jumping, walking, wing-fanning, walking in circles, walking on walls and ceilings, and walking around the light source or in zones with high light intensity due to reflections.

Contrary to the assumption of direct flight-to-light responses being a common and well-established moth response to light at night^8^, we did not find any evidence of such responses in our controlled lab experiment. However, a field study examining insect attraction to a light source reported that out of the 1,600 individuals observed to be affected by a light source, only 12 were actually caught at the light source itself^38^. Similarly, a recent study focusing on multiple moth species found that a mere 4% of the observed moths exhibited direct flight-to-light behaviour^39^. Our study supports the findings of Gaydecki^38^ and Degen et al.^39^, demonstrating that insects rarely exhibit a straightforward flight-to-light response as a result of phototaxis. The analysis of Gaydecki^38^ suggests that insects display more complex trajectory patterns at closer distances to the light source (40–50 cm), indicating potential disorientation. Samuel et al.^13^ conducted a study on insects’ 3D flight trajectories and discovered that insects tend to align their dorsum towards the light sources. In our study, although we did not use high-resolution cameras for recording, it would have been interesting to investigate whether moths in our experiments also exhibited this behaviour in response to the light source. Boyes et al.^8^ proposed that light at night directly affects moth’s phototaxis by distracting them and triggering negative phototaxis. However, our study uncovers a previously unobserved behaviour in which moths jump in front of the light, likely induced by high light intensity, disrupting their normal behaviour. To our knowledge, there are no existing studies that have documented this specific behaviour.

The influence of light at night on insect behaviour and activities remains unclear. Therefore, there is an urgent need for further studies on moth behaviour under controlled conditions. These studies will contribute to a better understanding of how the impacts on behaviour and activities in controlled settings relate to real-world field behaviour and long-term survival. Our experimental box, specifically designed and constructed for this study, offers a valuable tool for investigating the dose–effect relationships and behavioural responses of various species to different light levels or specific light sources.

## Supplementary Information

Supplementary Information 1.

Supplementary Video 1.

## Data Availability

All data generated or analyzed during this study are included in this published article (see Supplementary Datafile A and B).
