# Supplementary material for: Dose–effects in behavioural responses of moths to light in a controlled lab experiment
Source: Sci Rep. 2023 Jun 26;13:10339. doi: 10.1038/s41598-023-37256-0 (PMC10293237; doi:10.1038/s41598-023-37256-0)
Supplement: Supplementary file 1 — Supplementary Information 1. [file 41598_2023_37256_MOESM1_ESM.pdf]

Supplementary Figure S1. Appendix to Jägerbrand, AK, Andersson, P. Nilsson Tengelin M. Dose-effects in behavioural responses of moths to light in a controlled lab experiment

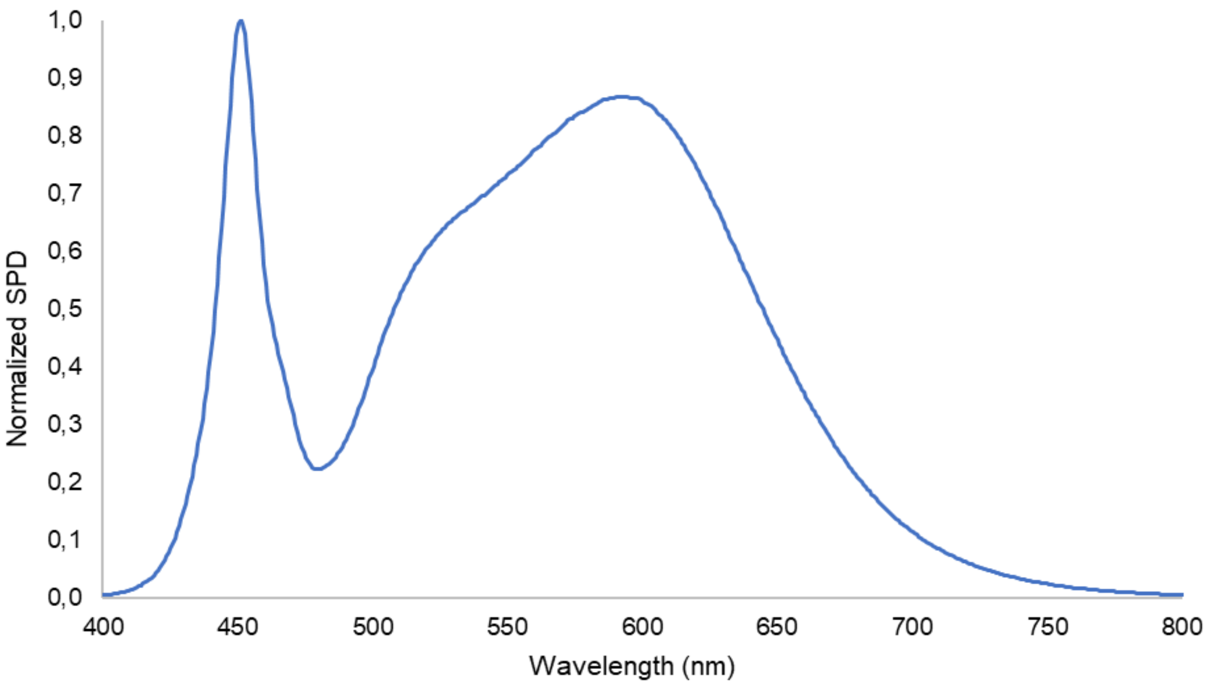

Figure S1. Normalized spectral power distribution of the LED light source used in the controlled lab experiment.

Supplementary **Datafile S1.** Appendix to Jägerbrand et al. Dose-effects in behavioural responses of moths to light in a controlled lab experiment.

							Number of jumps in the different zones in the box								
Light source luminance (cd/m2)	Sex (m=male, f=female)	Active time_ percent	NrJumps	Flown (Y=1, N=0)	On_lamp (Y=1, N=0)	NrOn Lamp	A	B	C	D	E	F	G	H	I
3,2	m	37,9	2	0	0	0		2							
19,52	m	80,4	0	0	1	1									
19,52	f	73,1	0	0	1	1								2	
640	m	97,5	1	1	1	2								1	
1280	f	75,8	3	1	1	1				1	2		1		
1280	f	95,5	11	1	1	7	2			5	2	2			1
0	m	43,8	0	0	0	0									
19,52	m	66,3	0	0	0	0									
19,52	m	26,2	0	0	0	0									
40	m	28,6	0	0	0	0									
80	m	92,4	4	0	1	2	2	2							
160	m	100,0	5	0	1	1	3	2							
640	m	13,3	0	0	0	0									
0	m	32,3	3	0	0	0			1	1			1		
0	f	22,7	0	0	0	0									

60,8	f	29,7	1	0	0	0		1							
60,8	m	10,5	0	0	0	0									
60,8	m	94,1	4	1	1	2	1	3							
60,8	f	60,2	8	0	0	0	5	1	2						
3200	m	29,0	0	0	0	0									
28,8	f	23,0	0	0	0	0									
28,8	m	12,2	0	0	0	0									
0	m	11,0	1	0	0	0							1		
320	m	37,4	13	1	1	1	1	3	3	6					
0		100,0	0	0	0	0									
160	f	63,3	16	0	1	1		2	1	6	6			1	
0	m	42,8	0	1	0	0									
160	f	52,7	5	1	1	1		2	1	2					
160	m	75,3	0	1	0	0									
0	m	18,5	0	0	0	0									
80	m	99,4	1	1	0	0			1						
80	m	82,9	14	1	1	4	10	4							
80	m	14,2	0	0	0	0									
640	f	20,8	2	0	0	0					2				
640	f	71,6	2	0	0	0					2				
0	m	88,3	1	0	0	0						1			
640	f	71,8	13	0	0	0	2	4	3	3	1				
1280	m	52,7	12	0	0	0	3	5	2		1	1			
1280	m	33,8	0	0	0	0									
1280	m	0,0	0	0	0	0									
1280	f	5,5	0	0	0	0									
320	f	100,0	0	0	0	0									
320	f	39,5	5	0	0	0					1	2	2		
320	m	55,4	14	1	1	6	3	6	3	2					
80	m	100,0	1	1	1	1		1							
40	f	18,8	3	0	1	1	1	1	1						
0	m	92,2	0	0	0	0									
40	m	87,3	0	1	0	0									
40	m	41,6	0	0	1	2									
40	f	25,3	13	1	0	0						4	3	4	3
0	m	82,0	0	0	1	1									
3200	m	5,5	0	0	0	0									
3200	m	99,8	1	1	1	2		1							
3200	m	33,8	3	1	1	1		1	2						
3200	m	71,0	114	1	0	0	1	21	35	23	7	8	19		
3200	m	0,0	0	0	0	0									
16	f	33,3	0	0	0	0									
16	m	61,2	1	1	0	0									1
16	m	2,2	0	0	0	0									
16	m	62,9	9	0	0	0				4	2	1	1	1	
0	m	9,3	0	0	0	0									
3,2	m	33,3	0	0	1	2									
3,2	m	47,2	0	0	0	0									
3,2	m	6,0	4	0	0	0									
3,2	f	52,5	1	0	0	0									

3,2	m	6,6	2	0	0	0		2					
3,2	m	47,5	0	0	0	0							
0	m	96,5	1	1	0	0						1	
0	m	8,6	11	1	0	0			1	6	4		
1,6	f	56,4	0	0	0	0							
1,6	f	55,8	0	0	0	0							
1,6	m	48,5	14	0	0	0							
1,6	m	14,7	0	0	0	0							
160	f	79,1	0	0	1	1							
1,6	m	16,5	0	0	0	0							
160	m	61,9	0	0	0	0							
160	m	16,5	0	0	0	0							
160	m	41,9	0	0	0	0							
160	m	6,8	2	0	0	0				1	1		
1280	m	12,2	0	0	0	0							
1280	m	100,0	1	1	1	3		1					
19,52	f	47,5	0	0	0	0							
3200		37,6	0	0	0	0							
28,8	m	6,5	0	0	0	0							
28,8	f	54,0	2	1	0	0						1	1
28,8	m	66,3	0	0	0	0							
0	m	22,0	0	1	0	0							
0	f	19,2	0	0	0	0							
2240	m	16,0	0	0	0	0							
2240	m	78,0	2	0	1	4	1				1		
2240	m	84,0	103	1	1	1	38	41	15	7	2		
2240	m	100,0	1	0	1	3			1				
0	m	100,0	0	1	1	1							
160	f	99,4	0	0	0	0							
0	f	74,6	0	0	0	0							
16	m	100,0	0	0	0	0							

Supplementary **Datafile S2.** Appendix to Jägerbrand et al. Dose-effects in behavioural responses of moths to light in a controlled lab experiment.

Light source luminance (cd/m2)	number of individuals	Sex (m=male, f=female)
0	17	m=13; f=3, unknown=1
1,6	5	m=3, f=2
3,2	7	m=6; f=1
16	5	m=4, f=1
19,52	5	m=3, f=2
28,8	5	m=3, f=2
40	5	m=3, f=2
60,8	4	m=2, f=2
80	5	m=5

160	10	m=6, f=4
320	4	m=2, f=2
640	5	m=2, f=3
1280	8	m=5, f=3
2240	4	m=4
3200	7	m=6, unknown=1
